# Inherent Importance of Early Visual Features in Attraction of Human Attention

**DOI:** 10.1155/2020/3496432

**Published:** 2020-12-22

**Authors:** Reza Eghdam, Reza Ebrahimpour, Iman Zabbah, Sajjad Zabbah

**Affiliations:** ^1^Faculty of Computer Engineering, Shahid Rajaee Teacher Training University, Tehran, Iran; ^2^School of Cognitive Sciences (SCS), Institute for Research in Fundamental Sciences (IPM), Niavaran, Tehran, Iran; ^3^Department of Computer, Torbat-e-Heydariyeh branch, Islamic Azad University, Torbat-e-Heydariyeh, Iran

## Abstract

Local contrasts attract human attention to different areas of an image. Studies have shown that orientation, color, and intensity are some basic visual features which their contrasts attract our attention. Since these features are in different modalities, their contribution in the attraction of human attention is not easily comparable. In this study, we investigated the importance of these three features in the attraction of human attention in synthetic and natural images. Choosing 100% percent detectable contrast in each modality, we studied the competition between different features. Psychophysics results showed that, although single features can be detected easily in all trials, when features were presented simultaneously in a stimulus, orientation always attracts subject's attention. In addition, computational results showed that orientation feature map is more informative about the pattern of human saccades in natural images. Finally, using optimization algorithms we quantified the impact of each feature map in construction of the final saliency map.

## 1. Introduction

Primates can detect conspicuous objects in cluttered scenes. Most of them can also move their eyes to different areas of the visual environment. They use this ability to move their fovea, the high resolution part of the retina, onto informative parts of the images. They focus on salient regions for more precise sampling of the information. Targeted eye movements provide them with an appropriate usage of processing resources for the most relevant visual information and a real-time perception of complex scenes [1].

Much research has been carried out on the underlying mechanisms of visual attention [2–7]. As one of the first studies, the importance of the local contrast on attention was investigated by Triesman and Gelade [8]. They defined a variety of features which can affect human attention. In their study, it was shown that there was no general feature which adequately contained salient content across all images. Koch and Ullman [9], using Feature Integration Theory, proposed the existence of an integrated map in the primate visual system that controls the region of interest in the visual field [9]. The proposed map prioritizes different regions of the visual scene for attention. The oculomotor system may use such map in order to move the eye toward the more prominent regions in the scene (overt attention (in an overt attention, subjects move their eyes to the attended region)). With respect to this theory, low-level feature extraction mechanisms act in a parallel manner over the entire visual scene. Extracted features are processed in different areas of the brain to provide the bottom-up cues towards the salient locations of the scene. In this context, the combined pooling of the basic feature maps is referred to as the saliency map. Many studies in this area have focused on observing the correlation (relation) between fixations made by human observers and the integrated saliency map [10].

Several computational models have been proposed to predict the salient regions of visual scene in order to simulate the visual attention of the primates [5, 11–15]. A frequently referred model of bottom-up attention is the model proposed by Itti et al. [13]. The biologically plausible approach of this model is laid on the contrasts of intrinsic low level visual features of images such as color, intensity, and orientation without any explicit usage of high-order features. Some studies have suggested other features or other computations for feature integration in order to improve the model's performance or make it more biologically plausible. Cerf et al. [16] added a face detection channel to the model, Itti et al. [17] added motion and flicker channels, Zabbah et al. [18] suggested a biologically plausible model for motion extraction, Torralba [19] modeled global scene factors, Tatler used a center-bias mechanism, Kootstra et al. [20] introduced symmetry as an effective source of attention, Parkhurst et al. [21] investigated the role of texture contrast, Bruce and Tsotsos [22], Li et al. [23], and Oliva et al. [24] used information theory for predicting salient regions of the natural images (for more details, see [1, 25]). Recently, the strength of deep neural networks to solve different tasks such as object recognition, object detection, and speech recognition have been shown in a wide range of studies. It has been proven that these networks can generalize well over different tasks [26]. In contrast to the classic models of bottom-up attention which mostly use low level features, models with deep structures incorporate high level features to predict human gaze map (Kümmerer et al. [27–30]), and they successfully reduced the gap between model prediction and ground-truth [31].

Evaluating these models, their final saliency maps are being compared with human eye locations in a free-viewing task. Many methods were defined to evaluate saliency maps such as Kullback–Leibler (KL) Divergence [32, 33], Normalized Scanpath Saliency (NSS) [12, 21], Area Under Curve (AUC) [11, 34], Correlation [35, 36], and Mutual Information [36].

Almost all attention models share a similar architecture and are organized into these three stages [11]:

1. Extraction: extracting different “feature channels” over the whole image plane
2. Activation: forming “activation maps” by processing on the feature channels
3. Normalization/Combination: normalizing and integrating the activation maps into a “final saliency map”

In the first stage, features such as color, intensity, orientation, and motion, are extracted from an input image. Then, in the next stage, some computational algorithm, which may be inspired from biology, is used to form the activation maps for each channel. Most of these algorithms have been implemented in a center-surround manner to establish concentric center-surround receptive field such as those in the brain. In order to simulate the receptive field of these neurons, models use Difference of Gaussian (DoG) filters [13, 37] or Pyramidal Gaussian Levels [38]. Finally, a normalization scheme is applied to activation maps which are in turn combined into a final saliency map.

A central problem in computational models of attention is that of combining feature maps into a final saliency map [1, 32, 39]. Knowledge on the effect of each basic feature in attraction of human attention may shed a light on the solution of this problem. The effect of basic features can inherently be different or can be controlled by top-down signals. Itti [40] investigated the contribution of low-level saliency in dynamic scenes and mainly focused on bottom up and top down attention. He showed that motion and flicker are better correlated with human saccades than color, intensity, and orientation, but not as good as all features combined. Frintrop et al. [41] designed a goal-directed model that weighted feature channels by using top-down cues that search for predefined objects in training dataset. Parkhurst et al. [21] also did some experiments on contribution of low-level factors in allocation of first saccade. In terms of inherent importance of basic features, Nothdurft [42] studied the role of orientation, motion, and color in preattentive attention. He reported a lack of importance of features in feature discrimination. Designing psychophysical experiments, he showed that the contrast is most informative feature for preattentive feature discrimination. However, this question still remained unanswered that which feature will first attract human attention when all present with the same detectability in an image simultaneously.

In this paper, we aimed to compare the inherent importance of low-level features in attraction of human attention. We investigated how different basic features compete with each other to attract human gaze. Our purpose was to find the inherent contribution of each feature in attraction of human attention. In psychophysical experiments, using simple synthetic images, we compared the probability of the attraction of attention in a co-presence of 100% detectable features. In addition, analyzing the output of two basic models of attention (Itti classic model [13] and GBVS model [43]) on four large dataset of natural images and human saccadic pattern on those images, we compared the ability of a single feature to predict human saccadic points. Using an optimization algorithm, we suggested a general static weight for feature combination in order to gain a better performance in prediction of human saccade landing points. These weights are not goal dependent and just depend on the nature of the features. The results were consistent with our psychophysics findings. Both results attributed a stronger role for orientation in the attraction of attention.

## 2. Material and Methods

We compared the effect of some early visual features (color, intensity, and orientation) on human visual attention. Designing psychophysics task and using some evaluation methods, we investigated which of these features may have more contribution in the visual attention. In the psychophysics task, we used simplified artificial stimuli to be able to control the feature strength. We made a competition between 100% detectable features in order to investigate whether there is an inherent importance in any basic feature to attract human attention. Using some evaluation methods on the feature maps of some attention models, the effects of these early features in natural images were considered. We used wide range of images in terms of their context to minimize the effect of context dependency.

### 2.1. Psychophysics Task on Synthetic Images

In this experiment, we aimed to find out whether the competition of 100% detectable feature for attraction of human attention has a special winner or not. Before designing a competition task between different modalities of features, we should be sure that each feature is 100% detectable in first saccade when it is present alone.

Our stimuli contained some red horizontal bars as distractors and one or more target (depend on the experiment phase) bars on a gray background. The gray background was chosen to ensure neutrality. Subjects were instructed to report the first target they detect. The positions of the targets were out of their fovea. Targets differed from distractors just in one basic feature: color, intensity, or orientation. The task had two phases performed by 15 subjects (21–42 years old, 7 females and 8 males). In the first phase, stimulus contained one target bar. This phase was designed to set the parameters of stimulus such as bar size, presentation time, and features' value in a way to make a same detectability for all features. The second phase was the main experiment. In this phase, stimulus contained more than one target with each differed in one feature. We investigated which feature will win the competition and attract human attention sooner.

**Figure 1:**
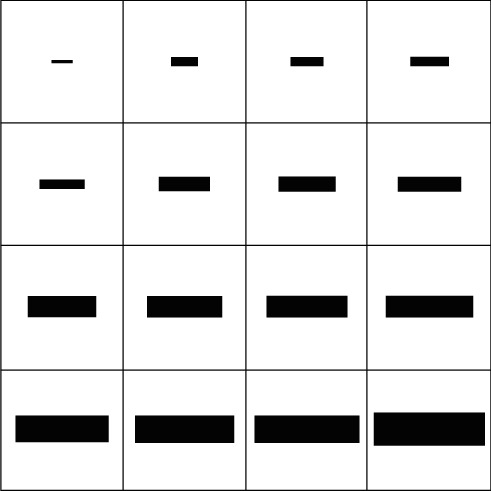
Size of bars in the control phase. Size of different bars that were used in the first phase (control phase) of psychophysics task is shown.

In the first phase, or so-called the control phase, each stimulus contained one target. This phase was implemented to make targets in different modalities 100% detectable. Choosing maximum contrast between a feature of target and distractors, we searched for a bar size and a presentation time which make the target of each modality 100% detectable. Proportion of correct responses averaged on all subjects and all trials is our measure of detectability. The size of bars and the presentation time spanned a range of 7 × 7 to 37 × 37 pixels in steps of two pixels and 100–300 ms in step 50 ms, respectively. The sizes of bars corresponded to those receptive field of cells in visual cortex (V1's simple and complex cells) which also were being used in computational models [44, 45].  Figure 1 illustrates the base bars that were used in the first phase for different features. There were three types of stimuli corresponding to the three different targets. In each type, target differed from distractors just in one feature. For each type of stimulus, a separate task was implemented to obtain the proper bar size and presentation time. So, we had three separated parts in the first phase. Below, stimuli which were used in each part will be described in detail.

**Figure 2:**
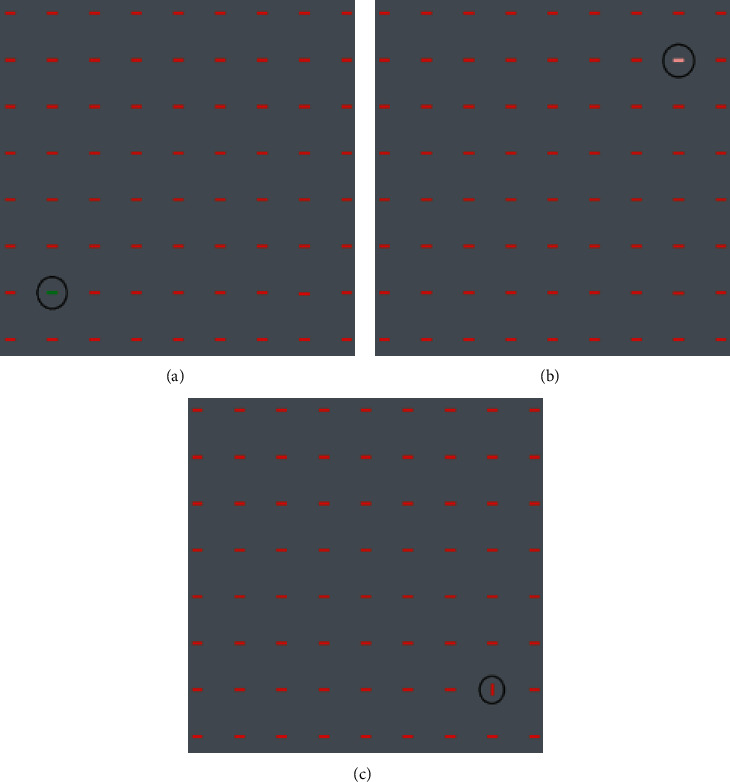
Stimuli of the first phase of psychophysics task. (a) Color, (b) intensity, and (c) orientation features.

**Figure 3:**
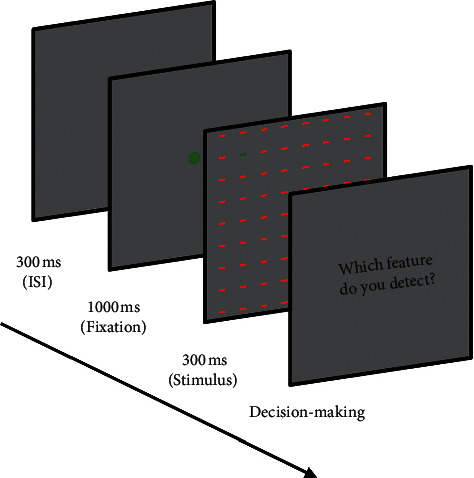
Psychophysics experiment procedure. For each stimulus, subjects were presented by 300 ms ISI, 1000 ms fixation point in center of screen, and 100–300 ms stimulus presentation; subjects reported whether they detected the target or not by pressing different keys on the keyboard. After subjects' response, the procedure was repeated.

For all stimuli, just one feature of the target was in strongest contrast with the distractors while other features were the same between target and distractors. For example, for the color feature, our stimuli contained a green horizontal bar as the target and some red horizontal bars as distractors (the target was the same as distractors in terms of intensity and orientation). The values chosen for color representation were taken from the opponent model (Red *vs.* Green) as implemented in attention models such as Itti. At the biological level, these opponent colors are coupled in neural representation in visual cortex [46]. For the intensity feature, the target bar had a color similar to distractors, but with an intensity near minimum (the target was the same as distractors in terms of orientation). For the orientation feature, in each stimulus, the target was located vertically among horizontal distractor bars (the target was the same as distractors in terms of intensity and color).  Figure 2 depicts three sample stimuli that were used in the three different parts of the first phase. For each size of bars (7 × 7 to 37 × 37 with step 2) and each presentation time (100–300 ms with step 50), we made 10 stimuli that were presented to subjects randomly. All stimuli had size of 900 × 900 pixels. The target bar was set in different random locations with 250–360 pixels far from the center of stimuli (where subject fixated) to be sure that the target is out of the fovea. Each presentation contained 300 ms ISI, 1000 ms a fixation point (center of screen), and 100–300 ms duration for stimulus. Participants were seated in a dark room, 50 cm away from a 19' CRT monitor connected to a computer [Intel Core i7 (2.4 GHz, 8 G RAM)] in resolution 1280 × 1024 pixels. We used MATLAB software (MathWork Inc., 2010) and stimuli were presented by the Psychophysics Toolbox [47]. Subjects were instructed to report as soon as they detect the targets by pressing related keys on the keyboard.  Figure 3 illustrated the procedure of stimuli presentation.

Results determined the proper bar size and presentation time which made the target bar 100% detectable. Since the detection of intensity did not reach to maximum performance, we declined this feature in the other parts of the experiment. The first bar size and stimulus presentation, by which all subjects could detect the target 100% correctly, were chosen for the next steps of the experiment.

**Figure 4:**
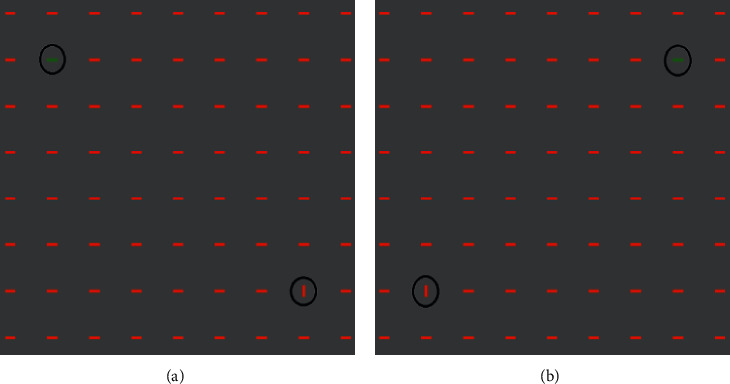
Stimuli of Competitive Attention task (CAT). This phase made a competition between the color and orientation targets, while the distractors had red color, the color target was green. In addition, the orientation target as red was the distractor but with different angels. This phase characterized which feature attracted the human attention first.

In the second phase, main experiment, we aimed to find the most salient feature for the first saccade in a competitive attention task (CAT). All stimuli contained two targets: orientation and color target bars (for details of why the intensity feature was discarded, see Section 3). This phase contained two parts. In the first part, features' value of the target set in the previous phase was used (vertical bar for the orientation target and green bar for the color target). In the second part, the angle of the orientation bar in different stimuli varied in the range of 0° (horizontal) to 90° (vertical) with steps of 10°. The targets were located symmetrically with the same distance from center of stimulus (as in the control phase). Thus, the targets were located on the opposite sides of a diagonal of a circle. The diagonal was selected randomly among all possible diagonal of a fix circle.  Figure 4 illustrates samples of the stimuli. The paradigm of the second phase was similar to the first phase. After 300 ms presentation of the stimulus, subjects were asked to report whether they detected the color target, the orientation target, both of them, or none of them. There were three keys labeled: color, orientation, and wave. They were instructed to press both the orientation and color keys for the detection of both targets and the wave key when they failed to detect any target. It should be noted that the timing of presentation allowed subjects to saccade just one time.

**Figure 5:**
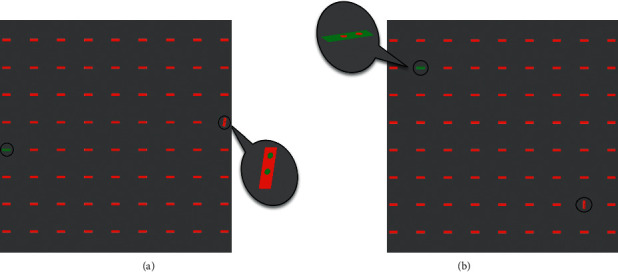
Stimuli of additional experiment of psychophysics task. In the second phase, an additional task was carried out to confirm results of CAT for overt attention. Stimuli in this experiment had target bars, which were marked with one, two, or three dots randomly. Dots were countable only if subjects make saccade onto them.

In order to confirm the obtained results for overt attentions, we designed another experiment in which we located one, two, or three dots in each target bar randomly and asked the subjects to report the number of dots beyond the type of the target (Figure 5). Dots were countable only if subjects make saccade to the target bar (overt attention). Results were quite similar to the main and control experiment.

### 2.2. Evaluation Methods with a Computational Approach on Natural Images

In order to measure the effect of each feature in the attraction of visual attention on natural images, we applied some evaluation and computational metrics on the feature and saliency maps that extracted from them. We used activation maps of Itti and GBVS models as feature maps for our experiments. Three feature maps (Color, Intensity, and Orientation) were computed and extracted for all images of four datasets. First and second stages of models were run on dataset to obtain feature maps, so our approach is focused on the third stage. Note that, in natural images usually all features with different strength compete with each other to attract human attention. In order to be independent of the context of images, we used four different datasets with different contexts. Here, we investigated which feature map had usually the maximum similarity with the human saccadic pattern for his first 5 saccades. Finally, we measured the importance of each feature map for predicting the human saccadic pattern for each image and also for each dataset.

In order to investigate the similarity of each individual feature with human saccadic map, we used correlation and mutual information (as linear and nonlinear measures of similarity). AUC (area under the ROC curve) were used to measure the ability of each single feature in prediction of human density map. To compute human density map, a Gaussian filter was applied on saccadic map which was obtained from eye-tracking data (for more information, see [11, 22]). Similarity measurements were also applied to feature channels to find more redundant ones. Finally, in order to quantify the effect of each feature map in the construction of the saliency map, we employed LSE (Least Square Error) and GA (Genetic Algorithm) to determine the weights of their linear combination with the aim of maximizing AUC between the combined map and the human density maps. Using LSE, we found the importance of each feature in each image, and while using GA, we found the importance of each feature for all images of a dataset.

#### 2.2.1. Dataset

Four dataset of natural images were used in the computational experiments. They are constructed by Judd et al. [48], Ehinger et al. [49], and Ramanathan et al. [50].

The Bruce & Tsotsos dataset (Toronto dataset) contains 120 natural images and saccadic eye-tracking data from 11 subjects. Images were presented for four seconds for eye-tracking data collection. All images had a size of 511 × 681 pixels (32° × 24°). For each image, saccades of all 11 subjects were collected and a binary saccadic map with the same size as the original images was made. The value of the pixels that were saccade landing positions were set to 1 and the rest were set to 0. After applying a Gaussian Filter on each saccadic map, human density map was being produced. These maps indicate the more probable positions for saccade [22].

The second dataset has been gathered by Ehinger et al. and consisted of 912 images of urban environments, half containing a pedestrian (target present) and half without (target absent). Images had a resolution of 800 × 600 pixels (23.5° × 17.7°). Participants were instructed to decide as quickly as possible whether a person was present in the scene. Eye movements of 14 observers were recorded [49].

The third dataset (MIT dataset) contains 1003 natural image (36° × 27°), which were observed by 15 subjects for 3 seconds. The dataset had saccadic eye-tracking data, but we used the abovementioned method to make human density map for all images [48].

The forth dataset contains 758 semantically images that are collected from Flicker, Photo.net, Google, and emotion-evoking IAPS. Images are in 1024 × 728 resolution (26° × 19°) and each of which was viewed by an average of 25 subjects for 5 seconds [51].

**Figure 6:**
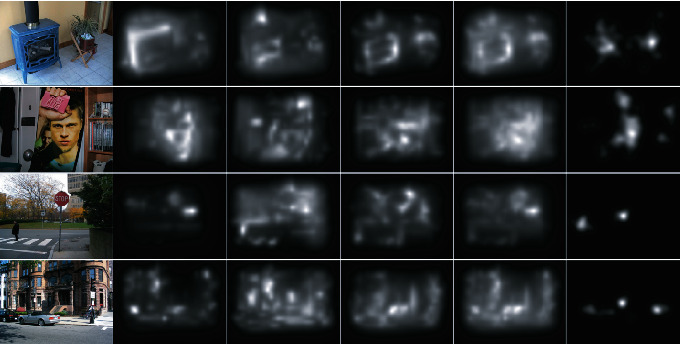
Dataset: some images of four datasets and their corresponding maps are shown. From left to right each row contained, respectively, original image, color feature map, intensity feature map, orientation feature map, final saliency map, and human density map.

Figure 6 shows some images of these dataset and corresponding feature and human density maps. First dataset had both saccadic eye-tracking data and human density map; but other three dataset had just eye-tracking data. So, for our experiments, we used the method mentioned in [11, 22] to construct human density maps.

#### 2.2.2. Correlation Method

In order to be able to compute the correlation between maps, we used 2D correlation [36]. 2D correlation between two images can be computed using the below equation:

(1) $$\begin{array}{c} C_{A,B}=\frac{\sum_{i=0}^{m-1}\sum_{j=0}^{n-1}a_{ij}b_{i,j}}{{\left\{\left(\sum_{i=0}^{m-1}\sum_{j=0}^{n-1}a_{i,j}^{2}\right)\left(\sum_{i=0}^{m-1}\sum_{j=0}^{n-1}b_{i,j}^{2}\right)\right\}}^{1/2}}, \end{array}$$

where *A* and *B* are the two images and *n* and *m* are the number of pixels of rows and columns. Here, *A* and *B* are different maps. We calculated correlation between two feature maps and between each feature map and human density map.

#### 2.2.3. Mutual Information Method

Mutual information is used in information theory as a measure of statistical dependence between two random variables. It measures the amount of information that one variable contains about the other one. In image processing, it can measure the ability of an image to explain another image [52]. The mutual information measure is computed as below:

(2) $$\begin{array}{c} MI\;\left(A,\;B\right)=H\left(A\right)+H\left(B\right)-H\left(A,\;B\right), \end{array}$$

where *H* is Shannon's Entropy [53], and for *m* given events occurring with probabilities *p*_*i*_ … *p*_*n*_, it is defined as follows:

(3) $$\begin{array}{c} H=\overset{m}{\underset{i=1}{\sum }}p_{i}\log\frac{1}{p_{i}}=-\overset{m}{\underset{i=1}{\sum }}p_{i}\log\;p_{i}. \end{array}$$

For an image, the entropy is calculated from the image intensity histogram in which the probabilities are the histogram values. It will have the maximum value if all levels of intensity have equal probability of occurrence and the minimum value (zero) if the probability of one level occurrence is 1 and the probability of all others occurring is zero. *H* (*A*, *B*) is joint entropy that can be calculated using joint histogram of two images. If the images are totally unrelated, then the joint entropy will be the sum of the entropies of individual images. The more similar the images are, the lower the value of the joint entropy is. In our analysis, mutual information determines the quantity of information that each channel has in common with other channels and human density map.

#### 2.2.4. Area under Curve

Area under curve (AUC) is the area under Receiver Operating Characteristic (ROC) curve. ROC is used in signal detection theory, medical decision-making, machine learning, and other scientific fields to show the evaluation of a binary classifier system as its discrimination threshold is varied [25, 54]. This criterion is widely used for measuring the performance of attention models. Here, we employed AUC to measure the performance of each feature channel in prediction of human density map and also to define a benefit function in GA.

#### 2.2.5. Least Square Error

Least Square Error (LSE) is a method to find optimum parameters which minimize the error between a predicted and desired signal [55]. Equation (4) shows the combination formulation of final saliency map using features, where *X* is the extracted feature map, *W* is the weight matrix, and *S* is the final saliency map. Each feature map is reshaped to a vector and all features together construct the matrix, *X*, or feature matrix which then are multiplied by a weight to construct the saliency map. Here, we want to find the weights in which *S* represents human density map. So, replacing *S* with human density map (equation (5)) and using the Least Square Error (LSE) method, optimal weights will be achieved through equation (6):

(4) $$\begin{array}{c} X_{\left[\left(n\text{Row}{}^{*}n\text{Col}\right)\times 3\right]}\times W_{\left[3\times 1\right]}=S_{\left[\left(n\text{Row}{}^{*}n\text{Col}\right)\times 1\right]}, \end{array}$$

(5) $$\begin{array}{c} X_{\left[\left(n\text{Row}{}^{*}n\text{Col}\right)\times 3\right]}\times W_{\left[3\times 1\right]}=D_{\left[\left(n\text{Row}{}^{*}n\text{Col}\right)\times 1\right]}, \end{array}$$

(6) $$\begin{array}{c} W_{\text{best}}={\left(X^{T}X\right)}^{-1}X^{T}D, \end{array}$$

where *n*Row and *n*Col are number of rows and columns of feature maps, respectively. The values of best weights can be interpreted as a quantity which explains the importance of each feature map on creating the best saliency map, that is, the most similar one to human density map for image *X*. Here, we look for the importance of each channel in final saliency map of each image. In other words, for each image, LSE will find a set of weights. Then, we can count the number of images in which, for example, orientation (or intensity or color) channel has most effect in their saliency map.

#### 2.2.6. Genetic Algorithm

Genetic Algorithm is an evolutionary algorithm that looks for one optimum point in a wide search space [56]. In our implementation, we searched for optimum weights of equation (4), defining the average of AUC of *S* (final saliency map) with respect to *D* (human density map) as the benefit function. We should note that, with the GA method, considering the average AUC of a dataset, we found a set of weights which can maximize the AUC of a whole dataset. In other words, we assume that there is a static weight for each channel which does not change image by image. In this approach, after optimization, GA suggests the best 3 weights in order to achieve the best AUC in whole dataset. Our GA starts with 60 random chromosomes as initial population. Each chromosome contains three genes corresponding to three different weights (weights of each channel). Algorithm applies the weights in each chromosome to equation (4) and calculates the AUC for each image. Averaging AUC across all images, 60 single AUC value corresponding to each chromosome will be achieved. Then, it selects 30 chromosomes which cause best AUC's as the parents of next generation. Using mutation and crossover as generation production methods second population will generate. The numbers of population in all generation is 60. The goal of the algorithm is to maximize the mean AUC of dataset. In order to avoid falling into local optima, some chromosomes of each generation reproduce randomly. In this way, algorithm obtained the optimum weights of feature channels for constructing a saliency map that follows human behavior in attention mechanisms.

## 3. Results

### 3.1. Psychophysics Results

In the control phase of psychophysics task as mentioned in material and methods, first we searched for a bar size and a presentation time which make the target bar 100% detectable (independent of target features). We found that targets in stimuli with bars in level 10 (25 × 25) and with 300 ms presentation time can be detected in 100% of trials. The detection of intensity target did not reach 100% in any sizes even for 300 ms presentation time. So, in the next step of the experiment, we used only color and orientation as targets.

**Figure 7:**
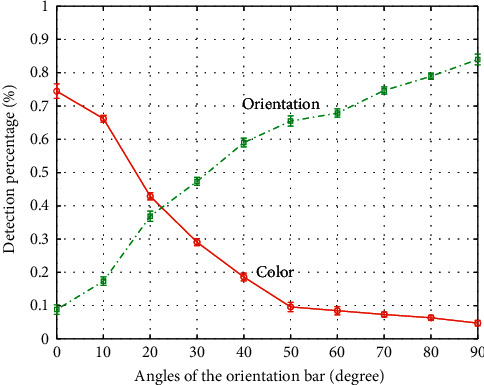
Results of competition task. Responses of subjects when stimuli contained both color and orientation features. Horizontal axis indicated the angle of orientation bars that changed from 0° to 90° with step 10°. Vertical axis showed the percentage of target detection.

In the second phase, we made a competition between color and orientation features as described in Section 2. All stimuli contained both color and orientation targets with their maximum contrast with distractors. In 84% of trials, orientation was detected as the first point of interest, while in less than 5% of trials, color won the competition. Other trials were those which subjects could not detect any target or rarely reported presence of both of targets. As illustrated in Figure 7, the stimuli in which the orientation target has the angle near to the horizontal bar, the color target can attract the attention of subjects more often. As the angles of orientation targets increased above 30°, the orientation target draws the attention of subjects more frequently and the detection of color target decreases exponentially.

### 3.2. Computational (Evaluation) Results

Psychophysical results showed that orientation feature was more effective in attraction of human attention in comparison with other features. In the computational part of our study, the impact of each feature in attracting human attention in natural images was investigated. Since natural images contain all features simultaneously, we can examine the effect of each feature in competition with other ones. For this goal, as mentioned in Section 2, we used correlation, MI, AUC, LSE, and Genetic Algorithm to determine the importance of each feature in prediction of the saliency map.

First, using correlation and mutual information, we checked the similarity between each feature map and human density map and also the similarity between each two feature maps. For each image, we computed three indicators for pairwise similarity between feature maps (Intensity *vs.* Color, Intensity *vs.* Orientation, and Color *vs.* Orientation) and three indicators for comparison of each feature map and human density map. We benefited the bar charts to show results. The length of the bars shows the percentage of images which takes the maximum value for that certain indicator. Then, using AUC, the contribution of each feature channel in the prediction of human saliency map (density map) was computed. Results show that how much each feature map can represent the human saliency map. Finally, we quantified the effects (weights) of different features in construction of the saliency map by LSE and GA methods. Although we used both Itti and GBVS models to extract basic visual features because of likeness of results, we just reported one of them (Itti model).

**Figure 8:**
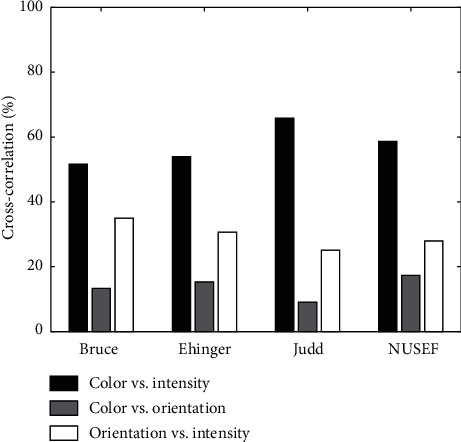
Correlation between features and density map. Horizontal axis indicates the four dataset used in computational experiments and vertical axis shows the percentage of images which correlation between each of their feature maps and human density map get the maximum value.

**Figure 9:**
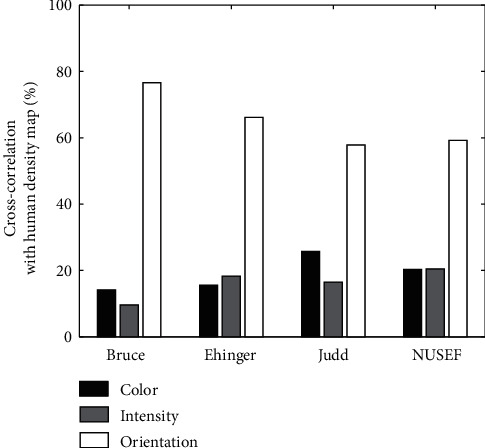
Correlation between features and correlation between two feature maps. Horizontal axis indicates the four dataset used in computational experiments, and vertical axis shows the percentage of images in which correlation between their feature maps gets the maximum value. The bars chart correlation between color and intensity, color and orientation, and intensity and orientation, respectively.

Correlation: correlation between each feature map and density map has been shown in Figure 8. In most of the images of all dataset, the correlation between the orientation and density map obtained the maximum value. There was approximately the same number of images for which the correlation of intensity and color maps with density map took the maximum values.  Table 1 presented the overall average correlation for each dataset between each feature channel and density map. As shown in Figure 9, color and intensity maps are usually the most correlated maps, while orientations and color maps are usually the least correlated maps.

**Figure 10:**
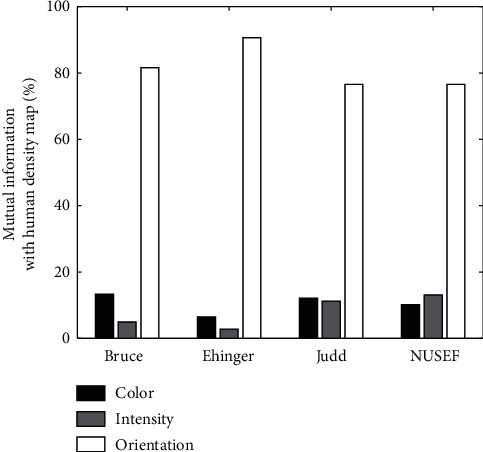
Mutual information between features and density map. Horizontal axis indicated the four dataset used in computational experiments and vertical axis showed the percentage of images which mutual information between each of their feature map and human density map get the maximum value.

**Figure 11:**
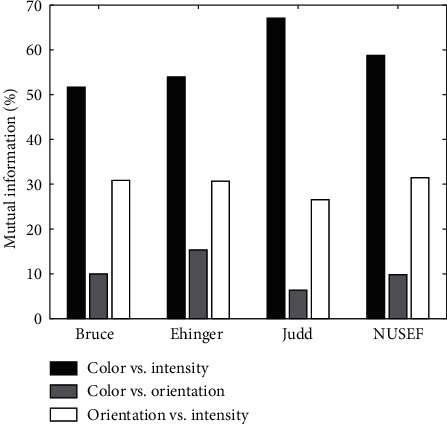
Mutual information between two feature maps. Horizontal axis indicated the four dataset used in computational experiments, and vertical axis shows the percentage of images which mutual information between their feature maps get the maximum value. The bars chart mutual information between color and intensity, color and orientation, and intensity and orientation, respectively.

Mutual Information: results of applying mutual information to the maps were presented in Figures 10 and 11, and Table 2. Here, we can also see that the color and intensity maps usually have most information in common. There are just a few number of images whose orientation and color maps have the most equivalent information. Similar to correlation we can see that, in most cases, orientation is the feature which carries the most information about density maps.

**Figure 12:**
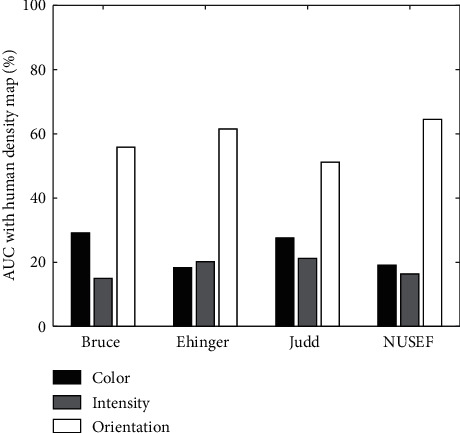
AUC between features and density map. Horizontal axis indicated the four dataset used in computational experiments, and vertical axis showed the percentage of images in which AUC between each of their feature map and human density map gets the maximum value.

AUC: AUC is widely used to evaluate the performance of attention models. Using this criterion, we evaluated the ability of each feature map to predict the human density map. Results show that, in most cases, the orientation map has a better AUC values (Figure 12). Also, the average AUC of the orientation map was always significantly greater than the other maps, as shown in Table 3. Interestingly, in some cases, orientation map has also a better performance than the overall saliency map computed by each model.

**Figure 13:**
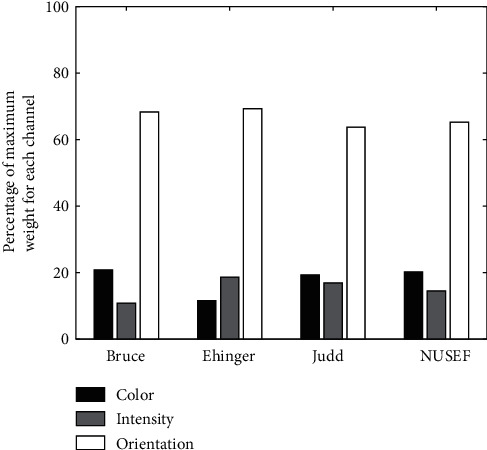
LSE. Percentage of images in which the weights for each of their feature maps gets the maximum value. For each image, three feature maps transformed to three vectors, density map transformed to a vector too, and then by using LSE, weight of each feature map for constructing saliency map (here density map) was computed. Number of maximum weights that maps take were counted over all images and reported in percentage.

LSE: we used LSE as an optimization method to obtain the optimal weights in a linear combination of feature maps, while the goal is to find a better representation of the human density map for each individual image. Results support the previous results obtained by correlation, mutual information, and AUC. The length of the bars in Figure 13 shows the number of images in which each corresponding feature map takes the strongest weight. Again the orientation map in most of the images takes the strongest weight. Overall averages of weights for each channel reported in Table 4. The orientation weight is significantly larger than weights of other features in all datasets.

GA: we also used genetic algorithm to optimize the weights in a linear combination of feature channels in order to find if there are static weights independent of the input image which can improve the overall AUC of a dataset. Obtained weights can be used in attention models as predefined static weights of each channel to simulate the effect of inherent importance of each channel. We ran our genetic algorithm 10 times and in all runs the same results were obtained.  Table 5 shows the optimal weights of feature channels that were obtained using genetic algorithm (averaged on 10 run). The orientation channel takes significant strongest weight in all datasets.

**Figure 14:**
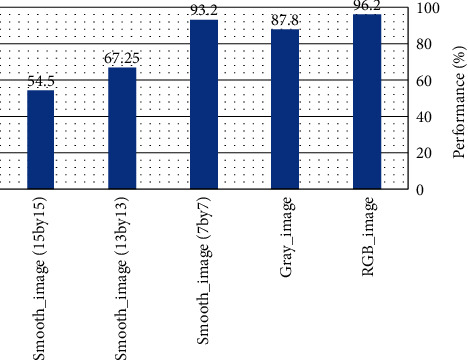
Performance of the AlexNet on a dataset under five different conditions. In the RGB_Image condition, images are colorful, in the Gray_Image condition, images are gray scale, and in the last 3 conditions, images were filters by an averaging filter with different sizes. Clearly, the AlexNet performance suffers more from diminishing of edges than color.

Deep structures: finally, we also tested how a deep structure behaves in the absence of color and edges information.  Figure 14 shows the categorization performance of the AlexNet on three categories (horse, butterfly, and cat) of the animal classification dataset [57]. The AlexNet was fine tuned on 2100 intact colorful images of the dataset (700 images in each category) and was tested on 90 new images (30 images for each category) in five different conditions. In the first condition, we calculated the accuracy of the model for the intact colorful images, the second one is the model accuracy for gray level images (where the color information was diminished), and the other three conditions are the accuracy of the model for smoothed images (where information about edges was diminish). We used three different averaging filters with the size of 7 × 7, 13 × 13, and 15 × 15 to decrease the strength of edges in the input image. As shown in Figure 14, the performance of the model is more affected by removing the edges' information (with sizes larger than 7 × 7) in comparison to color. This supports the hypothesis that orientation feature conveys more information than the color feature even when features are extracted with a deep structure.

## 4. Discussion

There are some studies which compared the role of early visual features [21, 41, 58] on the saliency map. They showed the effect of bottom-up attention in prioritizing a feature map. Nothdurft [42] showed that the contrast in each modality plays the most important role in prioritizing that modality. However, in this study, we investigated how simultaneous presentation of 100% detectable features affects the strength of each feature in the attraction of human attention. We showed that, although color and orientation target, with a specific contrast, could be detected in the first saccade, when they were appeared alone, orientation attracted human attention in the first saccade in nearly all trials when both features were appeared together.

## 5. Conclusion

In this study, we performed psychophysics tasks with synthetic stimuli and used some computational and evaluation metrics on the basic features of natural images to investigate the impact and contribution of early visual features in the attraction of human attention. In psychophysics, we first controlled the feature space to find 100% detectable feature. Then, we compare their detectability in a task in which both features were presented. Orientation almost always won the competition and attracted human attention in his first saccade. Moreover, using a wide range of natural images, we first consider the similarity between each feature map and human saccadic pattern or human density map. Orientation feature map was most informative map to predict human saccadic points. In addition, intensity and color maps were most similar maps, while information in orientation map was less predictable from other maps. This observation may tell us about the inherent importance of orientation feature in natural images. Taking psychophysics and computational results to account we may conclude that human attentional system prioritizes orientation feature because it has more information in comparison with intensity and color.

By using LSE and GA toward a linear combination of feature maps, we searched for the weight of each feature map in this integration. We used LSE to find the optimized weights per image. The goal was to achieve a best saliency map for each image. Then, we showed the number of images in which orientation or color or intensity weights get the maximum value. Results of the LSE method showed that, in most images, orientation weights get the maximum value. On the contrary, in the GA method the goal was to achieve the best average of AUC through the all images of dataset. So, GA found three static weights which can improve the average AUC of a whole dataset. Obtained weights can be used in attention models as predefined static weights of each channel to simulate the effect of inherent importance of each channel. In other words, using LSE, we saw that there are images in which other features (intensity and color) have stronger effect, but in order to achieve a better overall performance in a dataset (in terms of AUC) with three static weights, using GA, we showed that orientation weight should have a bigger value.

Although, it is believed that while familiar features compete strongly for saliency, different modalities contribute independently to the final saliency map, and our results suggest that different modalities also compete in the combination stage.

## Figures and Tables

**Table 1:** Correlation: average of correlation between each feature map and density map over all dataset's images. Rows are different datasets, and columns labeled color, intensity, and orientation show the average correlation between outputs of feature map of Itti model and human density map. Small columns show if the average of correlation of each map is significantly different with two other maps. NS stands for not significant (*p* > 0.05), *S* stands for significant difference (0.001 < *p* < 0.05), and HS stands for high significant difference (*p* < 10^−5^).

	Color	I	O	Intensity	C	O	Orientation	C	I
Bruce	0.3253	NS	HS	0.2949	NS	HS	0.5803	HS	HS
Ehinger	0.1421	S	S	0.1255	S	HS	0.2814	S	HS
NUSEF	0.0760	HS	HS	0.1988	HS	HS	0.3700	HS	HS
Judd	0.2157	HS	HS	0.2057	HS	HS	0.3215	HS	HS

**Table 2:** Mutual information. Average of mutual information between each feature map and density map over all dataset's images. Small columns show if the average of mutual information of each map is significantly different with two other maps. NS stands for not significant (*p* > 0.05), *S* stands for significant difference (0.001 < *p* < 0.05), and HS stands for high significant difference (*p* < 10^−5^).

	Color	I	O	Intensity	C	O	Orientation	C	I
Bruce	0.4742	NS	HS	0.4698	NS	HS	0.6856	HS	HS
Ehinger	0.4611	NS	HS	0.4794	NS	HS	0.6375	HS	HS
NUSEF	0.3012	NS	HS	0.3009	NS	HS	0.5684	HS	HS
Judd	0.2311	S	HS	0.2726	S	HS	0.4144	HS	HS

**Table 3:** AUC. Average of AUC between each feature map and density map over all dataset's images. Small columns show if the average of AUC of each map is significantly different with two other maps. NS stands for not significant (*p* > 0.05), *S* stands for significant difference (0.001 < *p* < 0.05), and HS stands for high significant difference (*p* < 10^−5^).

	Color	I	O	Intensity	C	O	Orientation	C	I	Saliency
Bruce	0.7993	NS	HS	0.7894	NS	HS	0.9211	HS	HS	0.9217
Ehinger	0.7513	NS	S	0.7442	NS	HS	0.8265	S	HS	0.8338
NUSEF	0.6677	S	S	0.7299	S	NS	0.7058	S	NS	0.6750
Judd	0.5997	HS	HS	0.7065	HS	HS	0.8109	HS	HS	0.7751

**Table 4:** LSE. Average weights of each unique feature map.

	Color	Intensity	Orientation
Bruce	0.1362	0.0561	0.4026
Ehinger	0.0987	0.0447	0.1923
Judd	0.0628	0.0023	0.1437
NUSEF	0.0141	0.0153	0.1724

**Table 5:** GA. Weights of feature channels that were obtained by applying genetic algorithm optimization on all images.

	Color	Intensity	Orientation
Bruce	0.2174	0.2036	0.5368
Ehinger	0.0742	0.0631	0.9110
Judd	0.3638	0.1129	1.0825
NUSEF	0.2736	0.2458	0.5967

## Data Availability

Both computational and psychophysical data are available from the corresponding author upon request.
